# Global prevalence of insulin resistance in the adult population: a systematic review and meta-analysis

**DOI:** 10.3389/fendo.2025.1646258

**Published:** 2025-08-22

**Authors:** Jhosmer Ballena-Caicedo, Fiorella E. Zuzunaga-Montoya, Joan A. Loayza-Castro, Juan Carlos Bustamante-Rodríguez, Luisa Erika Milagros Vásquez Romero, Rafael Tapia-Limonchi, Carmen Inés Gutierrez De Carrillo, Víctor Juan Vera-Ponce

**Affiliations:** ^1^ Instituto de Investigación de Enfermedades Tropicales, Universidad Nacional Toribio Rodríguez de Mendoza de Amazonas (UNTRM), Chachapoyas, Peru; ^2^ Facultad de Medicina (FAMED), Universidad Nacional Toribio Rodríguez de Mendoza de Amazonas (UNTRM), Chachapoyas, Peru; ^3^ Unidad de Posgrado, Universidad Continental, Lima, Peru

**Keywords:** insulin resistance, prevalence, public health, systematic review, meta-analysis

## Abstract

**Objective:**

To determine the global prevalence of IR, evaluating differences according to study designs and population characteristics.

**Methodology:**

A systematic review with meta-analysis was conducted. The search encompassed MEDLINE (PubMed), Scopus, Web of Science, and EMBASE, including observational studies that employed the HOMA-IR index to estimate IR and published adult prevalence data. Articles without clear IR definitions or with highly specific populations were excluded. The meta-analysis applied a random-effects model with proportion transformation (Freeman-Tukey), assessing heterogeneity with I² and Cochran’s Q test. Additionally, a meta-regression by publication year was conducted.

**Results:**

Eighty-seven studies were included, with 235,148 participants. The pooled prevalence of IR was estimated at 26.53% (95% CI: 24.10–29.03; I²=99%), with no statistically significant differences when comparing probabilistic versus non-probabilistic sampling or when stratifying by sex. The meta-regression revealed no clear variations according to publication year or other explored factors.

**Conclusions:**

This systematic review demonstrates that IR reaches a global prevalence of 26.53%, with estimated differences between 26% and 30% across different populations and geographical regions. Despite the diversity in cut-off points employed for HOMA-IR, no statistically significant differences were observed when comparing sampling designs or stratifying by sex. Furthermore, no clear trend related to publication year was evidenced.

## Introduction

Insulin resistance (IR) constitutes a highly relevant public health concern, as it is associated with metabolic disorders such as obesity, metabolic syndrome, and particularly type 2 diabetes mellitus (T2DM) (1). In brief, IR is defined as the decreased ability of peripheral cells to respond to insulin, resulting in altered glucose homeostasis and, over the long term, potentially predisposing individuals to chronic complications (2). In terms of magnitude, it has been estimated that more than 10% of the global adult population exhibits some degree of IR, with this figure varying according to factors such as ethnicity, age, and body mass index (3). Given the breadth of its clinical implications and the associated health impact, undertaking a systematic review that compiles and analyzes recent evidence on its prevalence is a priority.

Current trends show a sustained increase in IR and associated metabolic disorders, driven primarily by nutritional transition, physical inactivity, and population aging (4, 5). Genetic and epigenetic factors also contribute to this issue; however, the literature emphasizes the importance of unhealthy lifestyles as key triggers (6, 7). The growing number of overweight and obese individuals directly impacts the healthcare burden, increases healthcare costs, and affects individuals’ quality of life, additionally generating considerable socioeconomic impact (8). This situation demands an updated synthesis of the most recent research, enabling the establishment of evidence-based preventive and therapeutic action lines.

Despite the abundance of studies on IR, significant knowledge gaps persist related to the lack of homogeneity in diagnostic criteria, the diversity of measurement methods—primarily the IR index (HOMA-IR)—and population differences that hinder result comparability (9, 10). Additionally, some studies report discordant prevalences due to methodological differences and heterogeneity in cut-off point definitions, generating controversies regarding the true magnitude of the problem. These discrepancies highlight the need for a systematic and critical literature analysis to clarify the prevalence of IR and to unify criteria that would improve the quality and comparability of future studies.

The primary objective of this systematic review is to determine the prevalence of IR in the adult population. It also aims to evaluate its assessment methods and examine possible geographic and sex disparities. This objective’s scientific and clinical relevance lies in the need for robust and comparable data that guide health policies and evidence-based clinical practice guidelines. In doing so, this review will seek to fill the identified gaps regarding the variability of definitions and the lack of a clear picture of the true magnitude of IR at the global level.

## Methodology

### Research design

A systematic review with meta-analysis of studies evaluating IR prevalence was conducted, following the PRISMA guidelines (11) specifically adapted for prevalence research (12, 13).

### Search strategy

The literature search was conducted in four databases with broad coverage and relevance for epidemiological studies: MEDLINE (via PubMed), Scopus, Web of Science (including collections indexing SciELO), and EMBASE. These platforms were selected following Cochrane Collaboration recommendations for systematic reviews (14), given their extensive thematic scope, international recognition, and inclusion of studies from diverse geographical regions.

Key terms and MeSH descriptors employed included combinations of “insulin resistance” OR “insulin sensitivity,” “prevalence” OR “epidemiology,” among other relevant synonyms, combined with Boolean operators (AND, OR) and truncations according to each database’s requirements. A search interval up to March 1, 2025, was established to encompass the most updated evidence. The complete search equations and any additional details on the strategy employed in each database are available in **Supplementary Material 1**.

### Selection criteria

Observational studies (preferably cross-sectional) evaluating the prevalence of insulin resistance in adult populations (≥18 years), published between January 2000 and late January 2025, were included. Given the various methods for measuring IR, only those studies utilizing the HOMA-IR (Homeostasis Model Assessment of Insulin Resistance) test as the primary diagnostic criterion were selected, provided they explicitly indicated the cut-off point employed. Studies conducted in any geographical region and English or Spanish were considered.

Studies with highly specific populations whose primary objective was not to describe prevalence in a general adult population were excluded, as were narrative articles, letters to the editor, systematic reviews, bibliometric reviews, and case reports. Finally, studies presenting partial or inconsistent data, or those not allowing reliable quantitative information extraction on insulin resistance prevalence, were excluded.

### Study selection process

After applying the described search strategy, results obtained from the four databases were exported and imported into Rayyan software for effective reference management and automatic duplicate detection. Two independent reviewers (in blind mode) conducted an initial filter examining titles and abstracts based on the established inclusion and exclusion criteria. This initial phase allowed for discarding studies that, due to their subject matter, design, or absence of information on IR measurement, did not meet the minimum requirements for review. The delay time for this entire process was one and a half months.

Subsequently, articles passing this initial screening were evaluated in full text to confirm their eligibility. Discrepancies arising between the two reviewers were resolved by consensus, and when differences of opinion persisted, an independent third reviewer was consulted to settle the final decision. Once this process was concluded, a definitive list of included studies was developed, thus ensuring transparency and reproducibility of the selection at each stage.

### Data extraction

Two researchers independently extracted data using a standardized template designed in Microsoft Excel 2023. Bibliographic information (author(s), publication year), methodological details (study design, country or countries in which it was conducted, recruitment period or data collection), and relevant population characteristics (sample size, average age, sex proportion, and any significant sociodemographic data) were compiled. Likewise, sampling and recruitment methods (e.g., random sampling, recruitment in health centers) and diagnostic criteria or definitions used to identify IR were recorded, paying special attention to how HOMA-IR was measured and the specific cut-off point used in each study.

In addition to the main prevalence data, relevant secondary results were included, such as confidence intervals, the presence of comorbidities or associated risk factors, and any additional results that helped characterize the epidemiological profile of IR. Both researchers verified the extracted data’s consistency and resolved discrepancies by consensus to ensure information accuracy. When criterion differences persisted, an independent third reviewer was consulted to provide information fidelity in the final database.

### Risk of bias assessment

Two researchers independently conducted a risk of bias assessment using the Muun et al. tool (12) specifically developed for prevalence studies. This tool was chosen for its capacity to assess relevant domains in cross-sectional epidemiological investigations, such as sample representativeness, clear definition of the population at risk, precision in measuring the variable of interest, and identification of potential confounding factors.

After conducting the assessment, both researchers compared their results, and any discrepancy was resolved by consensus; if discordance persisted, intervention by a third reviewer was requested. In addition to the individual classification of each domain, a global score encompassing the sum of ratings was calculated, where studies with a score above seven were considered low risk, those obtaining between 4 and 6 points were classified as moderate risk, and those not exceeding 3 points were categorized as high risk. These assessments were incorporated into the final analysis to weigh the robustness of findings and facilitate discussion on the methodological quality of studies included in the synthesis.

### Statistical analysis

Quantitative analyses were conducted using R software (version 4.2.2), focusing solely on studies providing sufficient data on IR prevalence. Specifically, articles reporting the total number of participants (n) and the number of cases (r) with this condition were considered for meta-analysis. This approach allowed for consistent and comparable information, an indispensable requirement for obtaining reliable global prevalence estimates.

The pooled prevalence estimation was performed with the metaprop function from the meta package in R, applying the Freeman-Tukey proportion transformation (sm = “PFT”) to stabilize variance associated with prevalence data. Likewise, confidence intervals (CI) were determined through the Clopper-Pearson method (method.ci = “CP”), which was recognized for providing exact intervals for proportions. To address the expected heterogeneity between studies, a random-effects model was employed using the DerSimonian and Laird method (method.tau = “DL”), while the Hartung-Knapp approximation (hakn = TRUE) was applied to correct standard errors in the presence of significant variability.

The degree of heterogeneity was evaluated through Cochran’s Q test and the I² index, automatically estimated by the metaprop function. Forest plots were generated to visually illustrate the results, facilitating the comparison of point estimates and their confidence intervals. Meta-regressions were proposed to examine the influence of continuous factors, such as publication year and different HOMA-IR cut-off points, on prevalence estimates to delve into the causes of the observed variability. These meta-regressions were conducted using the rma function from the metafor package, employing mixed-effects models with weights inversely proportional to each study’s variance.

Additionally, a world map stratified by country and sex was developed to represent the geographical distribution of this event’s prevalence and to reflect possible demographic differences. To complement the analysis, bubble plots were generated, in which each bubble’s size corresponded to the statistical weight of the study within the meta-regression, thus offering a clear way to visualize each study’s contribution to the global estimate.

## Results

**Figure 1:**
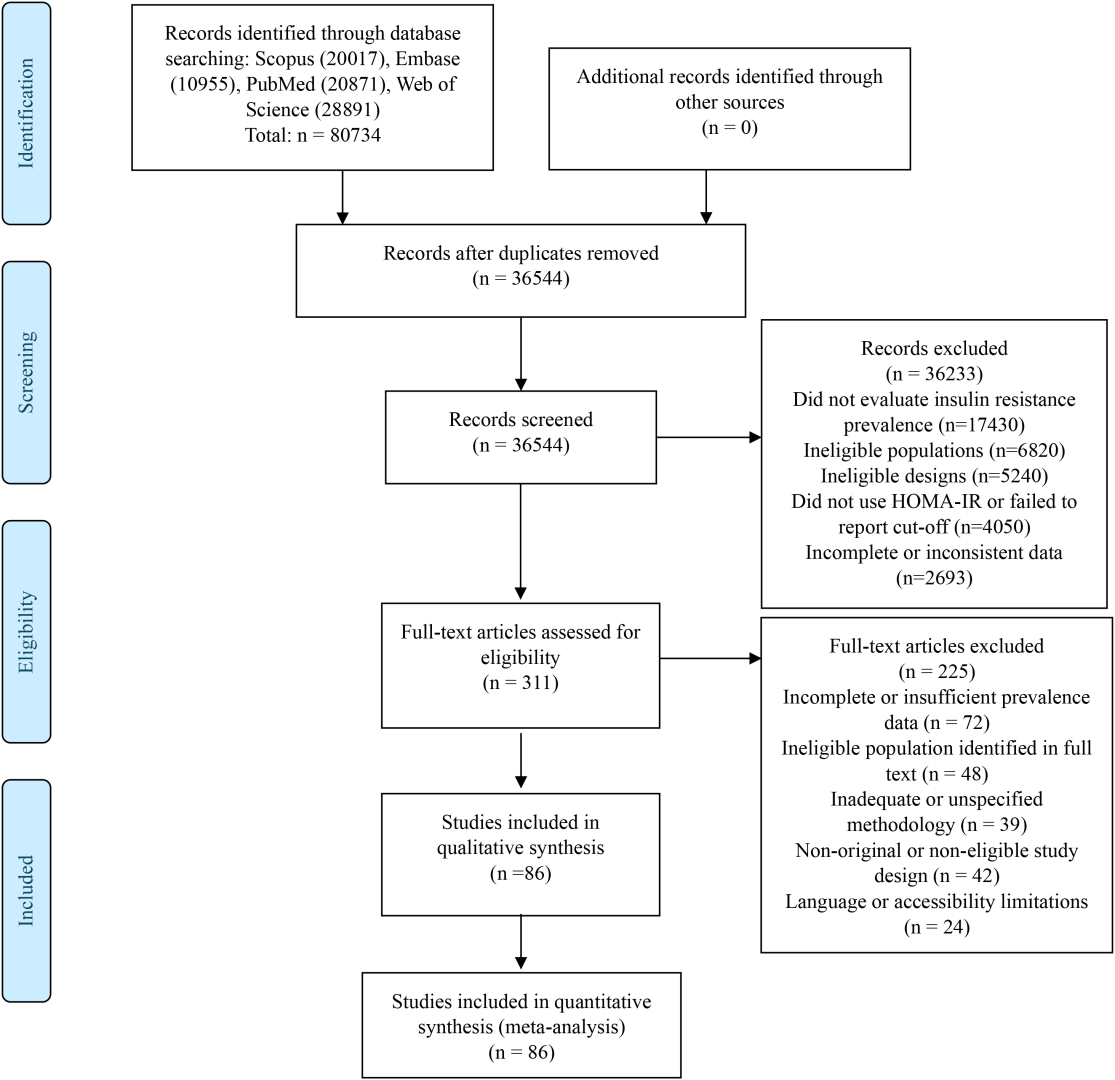
Flowchart of study selection.

**Figure 1** presents the PRISMA flow diagram summarizing the study selection process. A total of 80,734 records were identified through systematic searches in four main databases: Scopus (n=20,017), Embase (n=10,955), PubMed (n=20,871), and Web of Science (n=28,891). After duplicate removal, 36,544 records were screened by title and abstract. Of these, 36,233 were excluded, and 311 articles proceeded to full-text assessment. Finally, 86 studies met all inclusion criteria and were included in the qualitative synthesis and meta-analysis (15–100).

### General characteristics of included studies

Eighty-six studies published between 2001 and 2024 were included (**Supplementary Material 2**). Although scientific production remained relatively constant in the first decade (2001-2010), a notable increase was observed from 2011 onwards, with more than half of the publications concentrated in the 2011–2020 period. Geographically, a predominance of studies conducted in Asia was identified, highlighting the participation of China (7, 33, 36, 42, 52, 57, 63, 72, 73), South Korea (4, 22, 26, 28, 29, 37, 48, 65, 84), Japan (8, 25, 32, 54, 74), and Iran (18, 43, 58). In the same region, studies from Saudi Arabia (19), Kazakhstan (80), Lebanon (69), Qatar (71), Taiwan (7, 53), and Thailand (20) were included, configuring a diverse Asian block. In Europe, most works came from Spain (1, 3, 5, 9, 11, 13, 24), followed by Denmark (12, 15, 17, 27), Turkey (6, 59, 70), France (2), Georgia (40), Hungary (78), Italy (14), and Romania (78), and Hungary (78). America was represented primarily by the United States (21, 23, 30, 31, 35, 38, 76, 77, 81, 85, 86) and, to a lesser extent, by Mexico (45, 50, 82), Peru (55, 56, 60, 62, 64, 68, 83), Chile (10, 16), Brazil (75, 79), Argentina (39), and Venezuela (47). In Africa, studies from Nigeria (49, 61), South Africa (66), and Benin (34) were identified. Meanwhile, Australia was represented with two investigations (44, 51), and Belgium (46) completed the list of participating countries within Western Europe. Most articles were published in English, although a small percentage appeared in Spanish or other languages.

Only 10% of the investigations adopted a cohort approach (28–30, 49, 53, 59, 72, 98). Regarding sampling strategies, probabilistic methods predominated, including simple random sampling (15, 17, 24–26, 30, 31, 33) and cluster sampling (36), although several studies with non-probabilistic recruitment were also identified. Sample sizes presented wide variability, ranging from 86 participants to more than 21,000 in the most extensive samples. The median sample size was situated around 1,000 subjects, indicating considerable dispersion among studies.

The age ranges considered spanned from young adults (≥18 years) to older populations (≥65 years), with a predominance of studies including adults between 30 and 60 years. In terms of sex distribution, most works included men and women without sex segmentation (around 65%). At the same time, some focused exclusively on women (e.g., female population from universities or specific clinics) or men (mainly in occupational cohorts). Likewise, differences in recruitment context were observed: approximately half of the studies were developed in urban areas, a smaller percentage in rural areas, and the remainder combined both zones.

Although all selected studies used IR measurement as the primary variable, some heterogeneity in operational definitions was evident. While the HOMA-IR index was employed as the reference method in all works, cut-off points varied (generally between 2.0 and 2.7) according to guidelines established by each investigation. Some authors established a single value regardless of sex (e.g., ≥2.5 or ≥3.8), while others applied different criteria for men and women. Additionally, in most cases, participants with diagnosed diabetes mellitus, uncontrolled arterial hypertension, or medication use that altered glucose metabolism were excluded.

According to the assessment conducted using the selected tool, 83 studies (97%) showed a risk of bias categorized as low, while 3 (3%) were classified as moderate; no studies with a high risk of bias were recorded. Among the domains that presented greater compliance were the clarity of inclusion/exclusion criteria, precise determination of the primary variable, and detailed description of measurement procedures. Conversely, the most frequent limitations were related to sample representativeness in studies with non-probabilistic recruitment and the lack of information on response rate or losses during the sampling process. However, these deficiencies were detected in a small percentage of the works.

### Meta-analysis of IR prevalence and sensitivity analysis

87 studies evaluating the global prevalence of IR were included, with a cumulative total of 235,148 participants and a pooled prevalence of 26.53% (95% CI: 24.10–29.03). The observed heterogeneity was high (I² = 99%), indicating substantial differences between studies, such as evaluated population, selection methods, or diagnostic criteria. **Table 1** presents the overall global prevalence, whereas **Supplementary Material 3** provides the comprehensive forest plot showing each individual study.

**Table 1:** Results of the meta-analysis of IR prevalence according to diagnostic criterion, sampling design, and participants’ sex.

Variable	Number of studies	Number of participants	95% CI	I^2^
Global prevalence of IR	87	235148	26.53 (24.10 – 29.03)	99%
Sampling design				
Probabilistic	49	168633	26.90 (23.34 – 30.62)	100%
Non-probabilistic	38	66515	25.93 (23.45 – 28.49)	98%
Sex of participants				
Women	57	62598	27.67 (24.53 – 30.92)	98.7%
Men	57	57110	25.67 (22.98 – 28.46)	981%
HOMA-IR cut-off criteria				
≤2.5	46	150,218	27.96 (24.39 – 31.67)	99.6%
2.6-3.0	17	41,568	25.02 (19.21 – 31.33)	99.5%
>3.0	24	43,362	24.75 (21.96 – 27.64)	97.2%

Regarding sampling design, studies with probabilistic sampling (n=49) (18, 19, 22, 23, 27, 32, 34, 40, 42, 43, 45, 46, 51, 53, 54, 56, 59, 60, 62–64, 67–71, 73, 74, 76–78, 81–83, 85, 86, 94, 96) showed a pooled prevalence of 26.90%, while non-probabilistic ones (n=38) (18, 19, 22, 23, 27, 32, 34, 40, 42, 43, 45, 46, 51, 53, 54, 56, 59, 60, 62–64, 67–71, 73, 74, 76–78, 81–83, 85, 86, 94, 96) obtained a very similar estimate (25.93%). Regarding participants’ sex, results indicated a slightly higher prevalence in women (27.67%) (15, 16, 18–20, 22, 23, 25, 26, 28, 32–41, 45–56, 58, 60, 61, 64, 65, 67, 68, 70, 73–80, 82–84, 86–88, 90, 97, 98, 100) than in men (25.67%) (15, 16, 19, 20, 22, 23, 25, 26, 32–41, 45–56, 60, 61, 63–65, 67, 68, 70, 73–80, 82–88, 90, 97, 98, 100), although both subgroups also evidenced high heterogeneity (I² close to or above 98%) (See **Table 1**).

**Figure 2:**
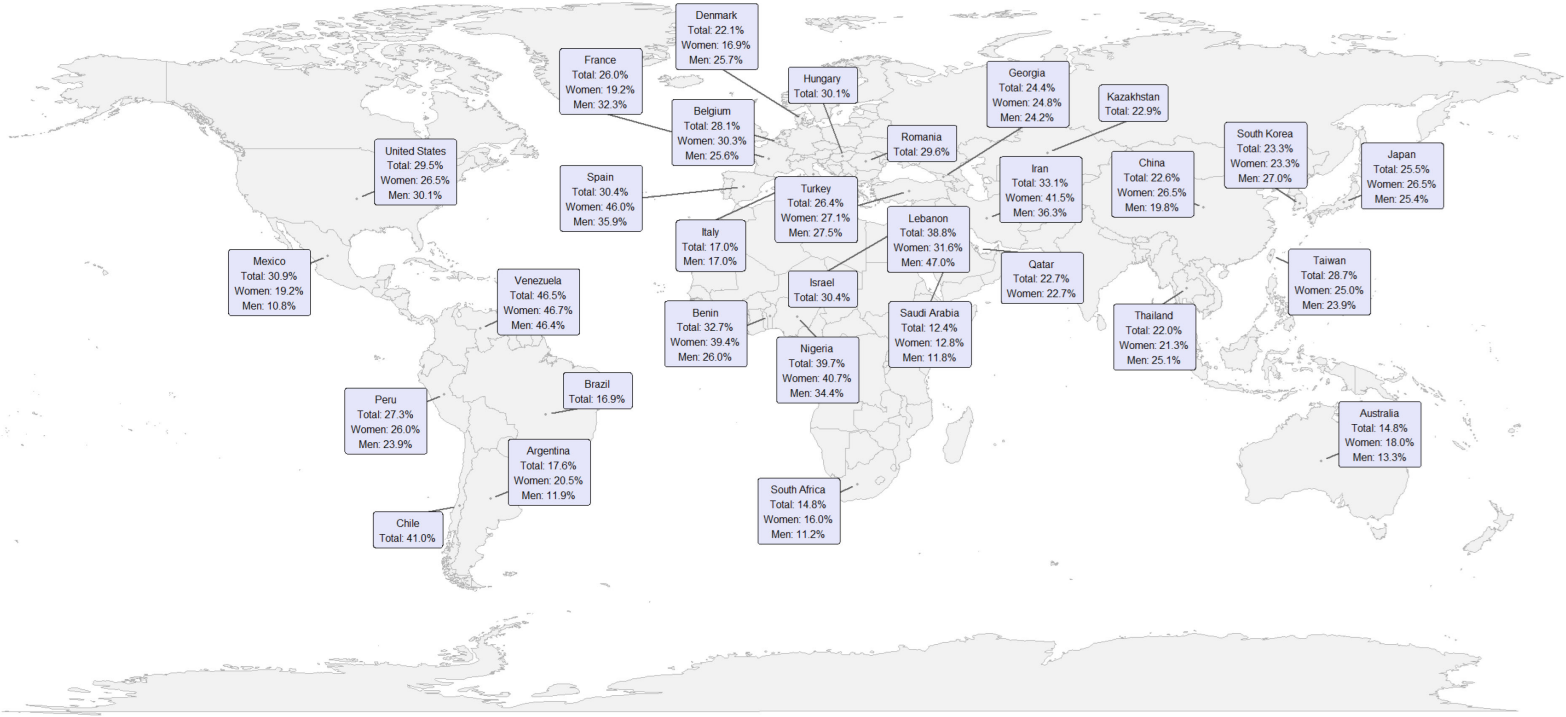
Global distribution of IR prevalence, and stratified by sex.

Additionally, the world map shows the distribution of IR prevalence in participating countries, presenting the total prevalence and that estimated by sex. Total estimates range from values close to 12.4% in Saudi Arabia (33) to around 46.5% in Venezuela (61), evidencing considerable variations between regions. Furthermore, it is observed that, in most national contexts, prevalence in women tends to be slightly higher than in men, although this difference is not homogeneous across all territories (See **Figure 2**).

Also, to address the heterogeneity in diagnostic criteria across studies, we performed a subgroup analysis stratifying results by HOMA-IR cut-off points (**Table 1**). Studies were categorized into three groups: liberal criteria (≤2.5, n = 46), moderate criteria (2.6-3.0, n = 17), and conservative criteria (>3.0, n = 24). As expected, lower cut-off points yielded higher prevalence estimates, with liberal criteria showing 27.96% (95% CI: 24.39-31.67), moderate criteria 25.02% (95% CI: 19.21-31.33), and conservative criteria 24.75% (95% CI: 21.96-27.64). Despite this stratification, substantial heterogeneity persisted across all subgroups (I² >97%), indicating that diagnostic threshold variability represents only one component of the observed heterogeneity. The liberal criteria group encompassed the largest number of studies (n = 46) and participants (n = 150,218), reflecting the predominant use of lower HOMA-IR thresholds in the literature.

### Meta-regression of IR prevalence by publication year

**Figure 3:**
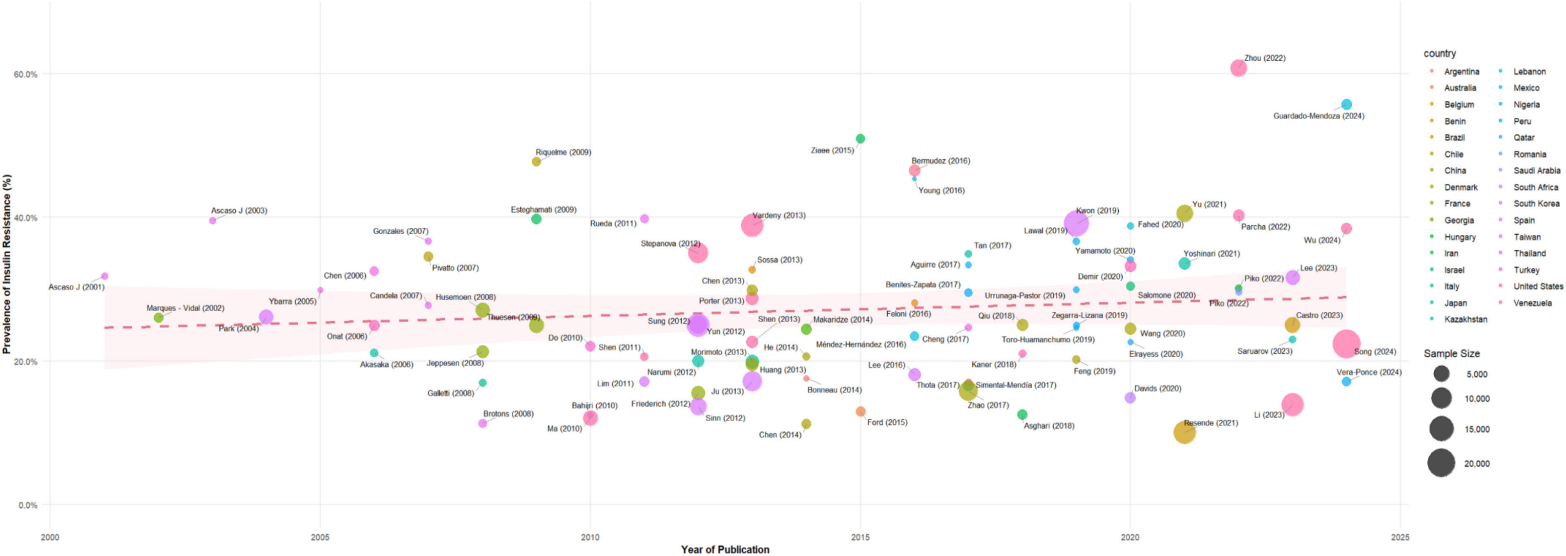
Meta-regression of IR prevalence by year and country.

The meta-regression based on publication year as a continuous variable shows a slightly ascending trend in IR prevalence over time, represented by the pink dotted line and its corresponding confidence band. Each point in the graph corresponds to a study included in the review, with bubble size proportional to sample size, allowing comparative visualization of the influence of participant volume on the point estimate of prevalence (See **Figure 3**).

Despite the model suggesting a moderate increase in IR rates over the years, the data exhibit considerable dispersion, evidencing that studies with similar publication dates may report diverse prevalences. Likewise, the participation of multiple countries is observed (indicated by different colors), reflecting the geographical breadth of the collected evidence and the possible influence of contextual factors on the variability of results.

### Assessment of publication bias

The funnel plot analysis (**Supplementary Material 4**) was conducted to evaluate potential publication bias in the included studies. The plot displays the Freeman-Tukey double arcsine transformed proportions on the x-axis against the standard error on the y-axis. The distribution of studies shows some asymmetry around the pooled estimate, with a concentration of studies near the center of the plot and some dispersion toward the extremes. While the funnel plot suggests the possibility of minor publication bias, the overall distribution pattern indicates that the majority of studies cluster around the pooled prevalence estimate, supporting the robustness of our meta-analysis findings.

## Discussion

### Main findings

This systematic review identified that insulin resistance is a highly prevalent condition in the adult population, with marked variability according to the diagnostic criterion used, especially regarding HOMA-IR index cut-off points. Despite this heterogeneity, general consistency in prevalence estimates was observed, without significant differences when comparing studies with different sampling designs or stratifying by sex. The meta-regression did not evidence a considerable trend related to publication year or other methodological characteristics, suggesting that differences between studies could be due, largely, to factors inherent to the population context and the lack of diagnostic standardization.

### Interpretation and comparison with literature

The findings of this systematic review, showing an average IR prevalence of around 26%–30%, are situated in an intermediate range when compared with results from previous investigations conducted in diverse geographic and population contexts. For example, in a recent meta-analysis centered on Southeast Asian countries (101), IR prevalence oscillated between 20% and 35%, which is in line with our general estimates. Other studies, including both clinical populations and broader population samples, also report comparable figures, such as the work by Li et al. (102), which found a significant association between elevated HOMA-IR values and greater prevalence of coronary calcification, not only supporting the relevance of IR as a generalized phenomenon but also underlining its cardiovascular implications.

Despite certain coincidences in global estimates, the literature evidences considerable disparities in IR prevalence, which can be attributed to various factors. First, diagnostic methodology presents important differences. Even when most works adopt HOMA-IR to estimate IR, there is no universal consensus on the optimal cut-off point (103, 104). This panorama is complicated by the utilization of specific values for each sex or the inclusion of populations with singular characteristics (e.g., women with PCOS or patients with subclinical hypothyroidism) in whom IR may be exacerbated (105, 106). Other reviews (103, 107) also insist on standardizing measurement methods and considering comorbid factors—such as obesity, sedentarism, or hormonal imbalances—that decisively influence prevalence.

Second, the sociodemographic and geographic context plays a determining role in the observed variability. Numerous studies point to dietary patterns, access to medical care, and genetic predisposition differing significantly between regions, translating into clinical and epidemiological heterogeneities (28, 101). For example, while in some East Asian cohorts, the cut-off point for HOMA-IR is located below 2 (20, 43), in Latin American studies, a threshold above 2.5 or even 3 (69, 70, 108) is often chosen. These methodological divergences can be amplified when evaluating populations of extreme ages—adolescents (103) or older adults (58)—or when considering associated comorbidities, such as metabolic syndrome and obesity (109), which alter the metabolic profile of participants.

Third, it is worth highlighting the statistical heterogeneity (elevated I²), an unequivocal sign of underlying differences between studies. While we have cut-off points and methodological criteria as an important point in these differences, the influence of behavioral and environmental factors, such as caloric intake, physical activity, and exposure to endocrine disruptors, is not always documented with the same intensity in all studies (42, 110, 110). Hence, at the population level, greater uniformity in sampling protocols and the collection of contextual variables is recommended, with the objective of more finely delineating the causes of heterogeneity (103).

Finally, it is relevant to highlight that while the HOMA-IR index is a useful and widely used instrument, it does not constitute the “gold standard” for IR measurement, which would be represented by the euglycemic-hyperinsulinemic clamp (111, 112). The ease of use and lower cost of HOMA-IR explain its popularity in large-scale epidemiological studies and its acceptable approximation to the reference method (113). Thus, IR emerges as a public health problem with high heterogeneity in its reporting and a strong multifactorial component, requiring coordinated efforts to standardize diagnostic criteria and delineate more effective preventive and therapeutic interventions.

Consequently, having an approximate measure of IR—although not as precise as the clamp—allows identifying risk groups not only with a greater risk of type 2 diabetes but also with cardiovascular disease (102), thyroid alterations (114), and hepatic complications (115). Thus, designing interventions aimed at modifying dietary habits, increasing physical activity, and preventing progression towards more severe metabolic conditions (116, 117). In this way, HOMA-IR is configured as a fundamental tool for clinical practice and research, provided its limitations are recognized and it is accompanied by standardized methodologies and unified diagnostic criteria.

### Implications for public health

The high prevalence of IR found in this SR underscores the magnitude of a problem that transcends the strictly clinical sphere and is configured as a public health priority. As previously mentioned, IR substantially increases the risk of various diseases, representing a relevant burden for health systems. Therefore, early detection of elevated IR values and implementation of prevention strategies in at-risk populations constitute essential measures to halt the advance of these chronic diseases.

In this sense, the existence of heterogeneous thresholds for HOMA-IR directly impacts health policies, as the disparity of diagnostic criteria may lead to underestimation or overestimation of real prevalence. For this very reason, to optimize epidemiological surveillance and IR screening, advancing towards greater standardization in diagnostic methodology is a priority, so that more uniform and internationally comparable clinical guidelines can be generated. A coherent diagnostic framework favors the development of comprehensive action plans, incorporating both preventive measures and treatment programs appropriate to each population group.

Moreover, the adoption of an index such as HOMA-IR in clinical practice and field studies—despite not being the “gold standard”—offers undeniable advantages from a public health perspective, especially due to its low cost and relative ease of use; since it allows early identification of individuals with greater metabolic susceptibility, health authorities could design interventions focused on lifestyle modification, such as promoting regular physical activity, improving nutritional quality, and reducing sedentarism. These interventions, primarily preventive in nature, are key to attenuating the growing incidence of diabetes and cardiovascular diseases associated with IR.

On the other hand, the results of this SR also show that IR can vary substantially according to sociodemographic, cultural, and environmental factors. Therefore, health policies must adapt to local realities, promoting community participation and collaboration between different sectors (such as education, agriculture, and urban development) to influence the social determinants of health. Multisectoral initiatives that address food security, availability of safe spaces for physical activity, and poverty reduction could have a significant impact on decreasing IR prevalence, especially in middle- and low-income countries.

Finally, given that IR is a risk factor transversal to multiple pathologies, its approach constitutes an opportunity to establish comprehensive health approaches. Strategies aimed at reducing IR could positively impact the prevention of various comorbidities and contribute to the sustainability of health systems by decreasing the economic and human burden associated with highly prevalent chronic diseases. Thus, the evidence gathered in this review reinforces the need to consider IR as a priority objective within public health agendas worldwide.

### Strengths and limitations of the review

One of this SR’s main strengths lies in the bibliographic search’s breadth, which encompassed multiple international databases, allowing the inclusion of studies from diverse geographical regions and population contexts. Likewise, standardized risk of bias assessment tools were employed, contributing to methodological rigor and transparency in the synthesis of results. The high number of total participants in the analyzed studies supports the statistical robustness of the aggregated estimates. Additionally, considering different cut-off points for HOMA-IR and the subgroup analysis according to sex and study design provides a detailed view of the variability in IR prevalence, offering valuable information for the formulation of health policies adapted to specific populations.

Among the most notable limitations is the high heterogeneity (I²) observed in the results, which hinders the direct comparability of estimated prevalences. This heterogeneity stems largely from the variability in recruitment methods, operational definitions of IR, and cut-off points employed for HOMA-IR. Finally, given the evolving nature of lifestyle factors, diagnostic criteria, and population demographics, future prevalence estimates of insulin resistance may differ from our current findings, particularly as new evidence emerges and populations undergo epidemiological transitions.

### Conclusions and recommendations

This SR demonstrates that IR reaches a global prevalence of 26.53% (95% CI: 24.10–29.03), with estimated differences between 26% and 30% across different populations and geographical regions. Despite the diversity in cut-off points employed for HOMA-IR, no statistically significant differences were observed when comparing sampling designs or when stratifying by sex. Furthermore, no clear trend related to publication year was evidenced.

In view of the methodological heterogeneity identified, standardization of IR diagnostic criteria is proposed through the adoption of consensus guidelines and the selection of cut-off points appropriate to each population. Likewise, implementing public health strategies based on nutritional education, promotion of physical activity, and prevention of obesity from early ages is recommended. Simultaneously, clinical practice and future research should prioritize the combined use of validated assessment methods, such as HOMA-IR and the euglycemic-hyperinsulinemic clamp, to improve diagnostic precision and comparability of results. Finally, the development of population screening programs is suggested, especially in groups with high metabolic risk, and the conduct of longitudinal studies to establish causal links and define more effective interventions in the prevention of IR.

## Data Availability

The original contributions presented in the study are included in the article/**Supplementary Material**. Further inquiries can be directed to the corresponding author.
