# Supplementary material for: Global prevalence of insulin resistance in the adult population: a systematic review and meta-analysis
Source: Front Endocrinol (Lausanne). 2025 Aug 22;16:1646258. doi: 10.3389/fendo.2025.1646258 (PMC12411212; doi:10.3389/fendo.2025.1646258)

Supplementary material 1. Search strategy

| **Database** | **Search Strategy** | **Filters Applied** |
| --- | --- | --- |
| **PubMed** | ("Insulin Resistance"[Mesh] OR "insulin resistance"[tiab] OR "resistencia a la insulina"[tiab] OR insulinoresistencia[tiab] OR "HOMA-IR"[tiab] OR "HOMA IR"[tiab] OR "HOMA index"[tiab] OR "HOMA test"[tiab] OR "homeostasis model assessment"[tiab]) AND "prevalence"[Mesh] OR prevalence[tiab] OR "epidemiology"[Mesh] OR epidemiology[tiab] OR frequency[tiab] OR "frecuencia"[tiab] OR "prevalencia"[tiab] | Humans, publication years 2000–2024, article type |
| **Scopus** | TITLE-ABS-KEY ( ( "Insulin Resistance" OR "insulin resistance" OR "resistencia a la insulina" OR insulinoresistencia OR "HOMA-IR" OR "HOMA IR" OR "HOMA index" OR "HOMA test" OR "homeostasis model assessment" ) AND ( prevalence OR incidence OR epidemiology OR frequency OR frecuencia OR prevalencia OR incidencia ) ) | Publication years >1999 and <2026  Document type: Article (ar)  Keyword filter: Human |
| **Web of Science (WOS)** | TS = ( "Insulin Resistance" OR "insulin resistance" OR "resistencia a la insulina" OR insulinoresistencia OR "HOMA-IR" OR "HOMA IR" OR "HOMA index" OR "HOMA test" OR "homeostasis model assessment" ) AND ( prevalence OR incidence OR epidemiology OR frequency OR frecuencia OR prevalencia OR incidencia ) | Publication years: 2000–2025  Document type: Article |
| **Embase** | 'insulin resistance'/exp OR 'insulin resistance' OR 'resistance, insuline' OR 'homeostasis model assessment'/exp OR 'homa' OR 'homa-ir' OR 'homeostasis model assessment' OR 'homeostasis model assessment insulin resistance' OR 'homeostasis model assessment of insulin resistance' OR 'homeostatic model assessment' OR 'homeostatic model assessment of insulin resistance' AND ('prevalence'/exp OR 'prevalence' OR 'prevalence study') | Years: 2000–2025  Limits: Humans and Articles only |

Supplementary material 2. Summary of the characteristics of the selected articles

| **First Author, Year** | **Country** | **Study Type** | **Sampling** | **Selection Criteria** | **Sample** | **Sex (% Female)** | **Age (Mean or Median in years)** | **Diagnostic Criteria** | **Cut-off Point** | **Risk of Bias Score** |
| --- | --- | --- | --- | --- | --- | --- | --- | --- | --- | --- |
| Ascaso J (2001) | Spain | Cross-sectional | Probabilistic | Volunteers 30-60 years with normal function. Excluded: history of dyslipidemia/diabetes/HTN, glycemia ≥110 mg/dl, hypertension, TG ≥150, CVD, obesity. | 292 | 165/292 (56.5%) | 43.9 | HOMA-IR/22.5 | ≥ 3.8 (3.9 in women and 3.5 in men) | 8 (low) |
| Marques-Vidal (2002) | France | Cross-sectional | Probabilistic | Subjects 35-64 years with fasting ≥10h. Excluded: insulin use. | 1153 | 556 (48.22%) | Men: 49.8 Women: 49.7 | HOMA-IR/22.5 | ≥ 3.8 | 8 (low) |
| Ascaso J (2003) | Spain | Cross-sectional | Probabilistic | Volunteers 25-65 years with normal function. Excluded: recent diet, weight change >10% in 3 months, hypothyroidism, insufficiency, neoplasms. | 283 | 153 (54.06%) | 47.26 | HOMA-IR/22.5 | ≥3.8 | 8 (low) |
| Park (2004) | South Korea | Cross-sectional | Non-probabilistic | Men ≥20 years. Excluded: medical diseases, glucose >126 mg/dl, HTN, medication use. | 4087 | ____ | ____ | HOMA-IR (Not specified) | >2.23 | 7 (low) |
| Ybarra (2005) | Spain | Cross-sectional | Non-probabilistic | No history of diabetes/hypertension/dyslipidemia, nor use of medications affecting metabolism. | 164 | 86 (53%) | Men: 41.8 Women: 41.6 | HOMA-IR (Not specified) | 3.8 | 7 (low) |
| Onat (2006) | Turkey | Cross-sectional | Probabilistic | Turkish cohort. Excluded: abnormal glucose regulation, non-fasting samples. | 1534 | 843 (54.95%) | ____ | HOMA-IR/22.5 | 2.245 | 8 (low) |
| Chen (2006) | Taiwan | Cross-sectional | Probabilistic | Participants >40 years. Excluded: use of lipid reducers. | 824 | 458 (55.58%) | 64.4 | HOMA-IR/22.5 | 1.35 | 8 (low) |
| Akasaka (2006) | Japan | Cross-sectional | Non-probabilistic | Medical controls Hokkaido 2001. Excluded: antihypertensive treatment, heart disease, diabetes. | 550 | 351 (63.81%) | 63.6 | HOMA-IR/22.5 | ≥1.73 | 7 (low) |
| Gonzales (2007) | Spain | Cross-sectional | Non-probabilistic | Population 40-75 years from Colloto. Excluded: immobilized, terminal, diabetics. | 305 | 172 (56%) | 55.5 | HOMA-IR (Not specified) | ≥ 2.9 | 7 (low) |
| Pivatto (2007) | Chile | Cross-sectional | Probabilistic | Born 1974-1978 in Limache. Excluded: diabetes treatment. | 996 | 556 (55.82%) | 24 | HOMA-IR (Not specified) | > 2.53 | 8 (low) |
| Candela (2007) | Spain | Cross-sectional | Probabilistic | Adults >30 years from Yecla. Excluded: pregnancy, foreigners, hyperglycemic treatments, serious diseases. | 292 | 149 (51.02%) | 52.8 | HOMA-IR/22.5 | ≥ 3.8 | 8 (low) |
| Husemoen (2008) | Denmark | Cross-sectional | Probabilistic | Inter99 cohort (1999-2001). Excluded: missing data. | 3609 | 1866 (51.7%) | ____ | HOMA-IR/22.5 | ≥ 1.77 | 8 (low) |
| Brotons (2008) | Spain | Cross-sectional | Non-probabilistic | Outpatients 18-79 years. Excluded: diabetics. | 690 | ____ | ____ | HOMA-IR (Not specified) | >3.5 | 7 (low) |
| Galletti (2008) | Italy | Cohort | Probabilistic | Normotensive male Olivetti workers. | 489 | ____ | 50.1 | HOMA-IR/22.5 | ≥ 2.77 | 8 (low) |
| Jeppesen (2008) | Denmark | Cohort | Probabilistic | MONICA Project 30-60 years. Excluded: history of infarction/stroke, without serum CRP. | 2357 | 1191 (50.53%) | ____ | HOMA-IR/22.5 | > 1.6 | 8 (low) |
| Riquelme (2009) | Chile | Cohort | Probabilistic | Adults ≥18 years Hispanic. Excluded: Amerindians, alcohol ≥20g/day, HCV+, CRP >10 mg/L. | 832 | 553 (66.5%) | 48.7 | HOMA-IR/22.5 | >2.6 | 8 (low) |
| Thuesen (2009) | Denmark | Cross-sectional | Probabilistic | Copenhagen cohort (1999-2006). Excluded: without follow-up. | 4516 | 2258 (50%) | 45 | HOMA-IR/22.5 | ≥1.76 | 8 (low) |
| Esteghamati (2009) | Iran | Cross-sectional | Non-probabilistic | Adults from three centers. Excluded: recent illness, pregnancy, hypertensives, smokers, <20 years. | 1276 | 838 (65.67%) | 38.4 | HOMA-IR/405 | 1.78 (1.69 for men and 1.81 for women) | 7 (low) |
| Bahijri (2010) | Saudi Arabia | Cross-sectional | Probabilistic | Subjects 18-50 years (2005-2007). Excluded: diabetes, endocrine disorders, hypertension, dyslipidemia. | 209 | 133 (63.6%) | 33 | HOMA-IR (Not specified) | >3.8 | 8 (low) |
| Do (2010) | Thailand | Cross-sectional | Non-probabilistic | Hospital staff >35 years Bangkok (2008). Excluded: antidiabetic use, diabetes. | 1217 | 990 (81.34%) | ____ | HOMA-IR/405 | Men≥1.56; Women: ≥1.64 | 7 (low) |
| Ma (2010) | United States | Cross-sectional | Probabilistic | NHANES 2005-2006. Excluded: without IgE results. | 4493 | 2337 (52%) | 46.8 | HOMA-IR/22.5 | ≥ 3.29 | 8 (low) |
| Lim (2011) | South Korea | Cross-sectional | Probabilistic | Adults ≥40 years with BMI 18.5-24.9 kg/m² from Chungju (2005-2006). Excluded: CVD, diabetes, HTN. | 1270 | 672 | ____ | HOMA-IR/22.5 | 1.74 | 8 (low) |
| Shen (2011) | United States | Cross-sectional | Probabilistic | NHANES 1999-2002, 20-39 years. Excluded: fasting <8h or >24h, diabetes. | 637 | 358 (51.9%) | 29.6 | HOMA-IR/22.5 | >3.233 | 8 (low) |
| Rueda (2011) | Spain | Cross-sectional | Probabilistic | Adults 18 years and older. Excluded: diagnosed diabetics. | 678 | 391 (57.66%) | 51.8 | HOMA-IR/22.5 | >1.24 | 7 (low) |
| Narumi (2012) | Japan | Cross-sectional | Probabilistic | Population ≥40 years from Takahata (2004-2005). Excluded: incomplete data. | 2200 | 1227 (55.77%) | 63 | HOMA-IR/405 | >2.5 | 8 (low) |
| Yun (2012) | South Korea | Cross-sectional | Non-probabilistic | Subjects ≥30 years. Excluded: CVD, cancer, extreme BMI. | 6430 | 2704 (42.05%) | Men: 48.9 Women: 48.8 | HOMA-IR/22.5 | ≥2.27 | 7 (low) |
| Friederich (2012) | Denmark | Cross-sectional | Probabilistic | Adults 19-72 years, Western Denmark. Excluded: missing data. | 3354 | 1847 (54.97%) | ____ | HOMA-IR/22.5 | ≥ 2.5 | 8 (low) |
| Sinn (2012) | South Korea | Cross-sectional | Non-probabilistic | Adults 30-59 years, BMI 18.5-24.9 kg/m². Excluded: excessive alcohol, diabetes, hepatitis, CVD. | 5878 | 3576 (61.8%) | 48.4 | HOMA-IR (Not specified) | ≥1.5 | 6 (moderate) |
| Sung (2012) | South Korea | Cross-sectional | Non-probabilistic | Examined 2003/2008 Samsung Hospital. Excluded: initial diabetes, missing data. | 12,853 | 3642 | ____ | HOMA-IR/22.5 | ≥2.0 | 7 (low) |
| Stepanova (2012) | United States | Cross-sectional | Probabilistic | Adults ≥18 years with complete data. Excluded: unconfirmed DM/IR, without HCV tests. | 9670 | 51.58 (4988) | 45.31 | HOMA-IR (Not specified) | ≥3.0 | 8 (low) |
| Porter (2013) | United States | Cross-sectional | Non-probabilistic | Subjects with abdominal CT and complete profile. | 2621 | 1305 (49.8%) | 51 | HOMA-IR/22.5 | > 3.06 for women; > 3.51 for men | 7 (low) |
| Morimoto (2013) | Japan | Cross-sectional | Non-probabilistic | Adults 30-69 years without initial diabetes. Excluded: missing data. | 3059 | 1305 (42.66%) | 55.7 | HOMA-IR/22.5 | >1.6 | 7 (low) |
| Chen (2013) | China | Cross-sectional | Probabilistic | Residents ≥18 years Wanzhai. Excluded: diabetes, missing data. | 1638 | 1021 (63.33%) | 51.98 | HOMA-IR/22.5 | >2.69 | 8 (low) |
| Sossa (2013) | Benin | Cross-sectional | Probabilistic | Subjects 25-60 years with complete follow-up. | 416 | 208 (50%) | ____ | HOMA-IR/22.5 | >3.2 | 8 (low) |
| Vardeny (2013) | United States | Cohort | Probabilistic | Caucasians/African Americans 45-64 years. Excluded: HF, diabetes, previous MI. | 12366 | 6913 (55.90%) | 54 | HOMA-IR/405 | ≥2.5 | 8 (low) |
| Huang (2013) | China | Cross-sectional | Probabilistic | Adults 20-74 years from Harbin. Excluded: vitamin D alterations, hepatitis, lipid medication. | 2708 | 1382 (51.03%) | 48.5 | HOMA-IR/22.5 | >2.50 | 8 (low) |
| Ju (2013) | South Korea | Cross-sectional | Non-probabilistic | Samsung Hospital Program (2009). Excluded: alcohol >20g/day, hepatitis, diabetes, smokers. | 9159 | 4107 (43.85%) | ____ | HOMA-IR/22.5 | >2 | 7 (low) |
| Shen (2013) | United States | Cross-sectional | Probabilistic | Adults 20-39 years with IFG/IGT. Excluded: diabetes, fasting <8h or >24h. | 2265 | 1211 (53.46%) | 29.5 | HOMA-IR/22.5 | >3.4351 | 8 (low) |
| Bonneau (2014) | Argentina | Cohort | Non-probabilistic | Detection Program Posadas, Argentina. Excluded: diabetes, hypo/hyperthyroidism, chronic diseases. | 176 | 117 (66.47%) | 49 | HOMA-IR/22.5 | ≥2.6 | 7 (low) |
| Makaridze (2014) | Georgia | Cross-sectional | Non-probabilistic | Georgian Caucasians 18-80 years (2012-2013). Excluded: diabetes, missing data. | 1522 | 653 (43%) | 45 | HOMA-IR (Not specified) | Women: ≥2.3; Men: ≥2.9 | 7 (low) |
| Chen (2014) | China | Cross-sectional | Probabilistic | Residents >18 years Wanzhai. Excluded: missing data, diabetes, obesity. | 1064 | 713 (67.01) | 50.82 | HOMA-IR/22.5 | >2.69 | 8 (low) |
| He (2014) | China | Cross-sectional | Non-probabilistic | Normoglycemics (FPG <6.1) from Chengdu. Excluded: medication altering IR/lipids, estrogens. | 533 | 230 (43.10%) | 62.8 | HOMA-IR/22.5 | ≥1.6 | 7 (low) |
| Ziaee (2015) | Iran | Cross-sectional | Probabilistic | Residents >20 years from Qazvin. Excluded: diabetes. | 982 | 502 (51.12%) | 39.2 | HOMA-IR/22.5 | >2.48 | 8 (low) |
| Ford (2015) | Australia | Cross-sectional | Probabilistic | Men >65 years (1996-2008). Excluded: without reassessment, without fasting, missing data, depression, diabetes. | 1162 |  | ____ | HOMA-IR (Not specified) | ≥1.85 | 8 (low) |
| Méndez-Hernández (2016) | Mexico | Cohort | Non-probabilistic | Health workers 20-70 years from Mexico. Excluded: incomplete data, CVD, cancer, IR, pregnancy. | 956 | 717 (75%) | 53.3 | HOMA-IR/22.5 | >3.2 | 7 (low) |
| Feloni (2016) | Belgium | Cross-sectional | Non-probabilistic | Adults 20-80 years preoperative. Excluded: diabetes, without HOMA-IR, without waist circumference. | 288 | 155 (54%) | 55 | HOMA-IR/22.5 | ≥3 | 7 (low) |
| Bermudez (2016) | Venezuela | Cross-sectional | Probabilistic | Participants from Maracaibo with consent. | 2026 | 1056 (52.10%) | 39.69 | HOMA-IR (Not specified) | ≥ 2 | 8 (low) |
| Lee (2016) | South Korea | Cross-sectional | Non-probabilistic | Adults 40-79 years with brain MRI. Excluded: previous stroke. | 2326 | 1047 (45%) | 56.2 | HOMA-IR/405 | ≥2.56 | 7 (low) |
| Young (2016) | Nigeria | Cross-sectional | Non-probabilistic | Women >18 years religious conference. Excluded: pregnancy, lactation, chronic diseases. | 86 | 86 (100%) | 44.4 | HOMA-IR (Not specified) | >2 | 7 (low) |
| Simental-Mendía (2017) | Mexico | Cross-sectional | Non-probabilistic | Healthy adults 18-23 years from Mexico/Durango. Excluded: alcohol ≥20g/day, tobacco, overweight, hepatitis. | 1732 | 1187 (68.53%) | 19.1 | HOMA-IR/22.5 | >2.5 | 7 (low) |
| Thota (2017) | Australia | Cross-sectional | Probabilistic | Adults ≥65 years RHLS. Excluded: diabetes, glucose ≥7 mmol/L, medications. | 482 | 283 (58.23%) | 77.78 | HOMA-IR/22.5 | ≥2.5 | 8 (low) |
| Zhao (2017) | China | Cross-sectional | Probabilistic | Adults ≥18 years residents ≥6 months in China. Excluded: missing data, hepatitis, medication. | 8398 | 4984 (59.3%) | 52.16 | HOMA-IR/405 | >1.6 | 8 (low) |
| Cheng (2017) | Taiwan | Cross-sectional | Non-probabilistic | Adults 50-90 years from Taoyuan. Excluded: missing data, extreme values. | 394 | 256 (65%) | 64.4 | HOMA-IR (Not specified) | ≥2.3 | 7 (low) |
| Tan (2017) | Japan | Cross-sectional | Non-probabilistic | 75g OGTT (2001-2009) in Rumoi. Excluded: ischemic heart disease, anticoagulant therapy, missing data. | 404 | 207 (51.23%) | 63.2 | HOMA-IR/405 | ≥1.7 | 7 (low) |
| Benites-Zapata (2017) | Peru | Cross-sectional | Non-probabilistic | Adults ≥18 years without diabetes/metabolic diseases. Excluded: glucose ≥126 mg/dl, corticosteroids. | 600 | 401 (67%) | 36.8 | HOMA-IR/405 | ≥3.8 | 7 (low) |
| Aguirre (2017) | Peru | Cross-sectional | Non-probabilistic | Adults >18 years without diabetes/metabolic disorders. Excluded: ≥60 years, altered glucose/hormones. | 213 | 137 (64.3%) | 35.8 | HOMA-IR/405 | ≥3.8 | 7 (low) |
| Qiu (2018) | China | Cross-sectional | Non-probabilistic | Mongolians ≥20 years. Excluded: CVD, endocrine diseases, antihypertensive use, diabetes. | 1903 | 1168 (61.40%) | ____ | HOMA-IR/22.5 | ≥ 3.28 | 7 (low) |
| Asghari (2018) | Iran | Cohort | Probabilistic | Adults ≥20 years. Excluded: previous MI/stroke, abnormal energy intake, pregnancy. | 1205 | 687 (57%) | 42.7 | HOMA-IR/22.5 | > 3.2 | 8 (low) |
| Kaner (2018) | Turkey | Cross-sectional | Non-probabilistic | Women 20-49 years in İzmir clinics. Excluded: pregnancy, lactation, menopause, underweight, diabetes. | 527 | 527 (100%) | ____ | HOMA-IR/405 | ≥2.7 | 7 (low) |
| Toro-Huamanchumo (2019) | Peru | Cross-sectional | Non-probabilistic | Adults >18 years normal BMI without diabetes/hypothyroidism. Excluded: ≥60 years, altered glucose. | 118 | 97 (82.20%) | 37.5 | HOMA-IR/405 | ≥2.28 | 7 (low) |
| Lawal (2019) | Nigeria | Cross-sectional | Probabilistic | Subjects 18-70 years without diabetes. Excluded: pregnancy, illness, glucocorticoid use. | 396 | 207 (52.27%) | 40.4 | HOMA-IR/22.5 | >2.2 | 8 (low) |
| Urrunaga-Pastor (2019) | Peru | Cross-sectional | Non-probabilistic | Adults >18 years euthyroid without diabetes Lima (2012-2016). Excluded: ≥60 years, altered glucose. | 204 | 164 (81.4%) | 38.5 | HOMA-IR/405 | ≥ 3.8 | 7 (low) |
| Feng (2019) | China | Cross-sectional | Non-probabilistic | Residents Chengdu. Excluded: insufficient insulin data, diabetes. | 570 | 250 (43.85%) | 62.3 | HOMA-IR/22.5 | ≥1.66 | 7 (low) |
| Zegarra-Lizana (2019) | Peru | Cross-sectional | Non-probabilistic | Adults 18-60 years normal BMI Lima. Excluded: pregnancy, corticosteroids, endocrinopathies. | 284 | 254 (88.1%) | 33.77 | HOMA-IR/405 | >2.32 | 6 (moderate) |
| Kwon (2019) | South Korea | Cross-sectional | Probabilistic | KNHANES 30-59 years (2007-2010). Excluded: fasting <8h, excessive alcohol, diabetes, liver disease. | 16371 | 10385 (63.43%) | ____ | HOMA-IR/405 | >2.18 for men and >2.19 for women | 8 (low) |
| Davids (2020) | South Africa | Cross-sectional | Probabilistic | Residents Cape Town ≥18/20 years (2008-2016). Excluded: bedridden, pregnancy, underweight. | 1706 | 1278 | 48.2 | HOMA-IR (Not specified) | >3.1 | 8 (low) |
| Salomone (2020) | Israel | Cross-sectional | Non-probabilistic | Adults 40-70 years Tel Aviv (2010-2015). Excluded: hepatitis, excessive alcohol, extreme calories. | 789 | 374 (47.4%) | 58.83 | HOMA-IR (Not specified) | >3.31 | 7 (low) |
| Yamamoto (2020) | Peru | Cross-sectional | Non-probabilistic | Adults 18-60 years Lima (2014-2016). Excluded: elevated glucose/HbA1c, thyroid alterations. | 261 | 199 (76.3%) | 39 | HOMA-IR/405 | ≥ 3.8 | 6 (moderate) |
| Fahed (2020) | Lebanon | Cross-sectional | Non-probabilistic | Notre Dame employees (Zouk Mosbeh). Excluded: pregnancy, CVD, diabetes, pacemakers. | 286 | 152 (52.1%) | 41.2 | HOMA-IR/22.5 | ≥ 2.5 | 7 (low) |
| Demir (2020) | Turkey | Cross-sectional | Probabilistic | Adults ≥20 years Tokat. Excluded: refusal, not located. | 2013 | 1139 (56.6%) | 47.2 | HOMA-IR (Not specified) | 1.4 | 8 (low) |
| Elrayess (2020) | Qatar | Cross-sectional | Non-probabilistic | Female university students 20-79 years. | 150 | 100% | 22.4 | HOMA-IR (Not specified) | >1.85 | 7 (low) |
| Wang (2020) | China | Cross-sectional | Non-probabilistic | Rural residents ≥40 years without stroke/TIA Shandong. Excluded: missing data, MRI contraindication. | 2007 | 1050 (52.31%) | ____ | HOMA-IR/22.5 | ≥3.0 | 7 (low) |
| Yu (2021) | China | Cross-sectional | Probabilistic | CHNS >18 years. Excluded: pregnancy, missing data, diabetes, CVD, kidney/liver disease. | 6027 | 3184 | ____ | HOMA-IR/22.5 | ≥2.5 | 8 (low) |
| Yoshinari (2021) | Japan | Cross-sectional | Probabilistic | Hisayama residents 40-79 years. Excluded: without consent, diabetes, without OGTT. | 2094 | 1214 (58%) | 60 | HOMA-IR/405 | ≥1.61 | 8 (low) |
| Resende (2021) | Brazil | Cross-sectional | Probabilistic | Employees 35-74 years (2008-2010). Excluded: inconclusive Chagas, bariatric surgery, diabetes. | 12348 | ____ | ____ | HOMA-IR/405 | 90th percentile | 8 (low) |
| Parcha (2022) | United States | Cross-sectional | Probabilistic | NHANES 18-44 years. Excluded: pregnancy, lactation, CVD, IR, missing data, diabetes. | 1877 | 876 | ____ | HOMA-IR/405 | ≥2.5 | 8 (low) |
| Piko (2022) | Hungary | Cross-sectional | Probabilistic | Adults 20-64 years with consent. Excluded: missing phenotype/genotype data. | 372 Hungary, 334 Romanian | Hungary: 278 (74.6%), Romanian: 183 (54.8%) | Hungary: 42.60, Romanian: 44.20 | HOMA-IR (Not specified) | >3.63 | 8 (low) |
| Zhou (2022) | United States | Cross-sectional | Probabilistic | NHANES >18 years (2011-2016). Excluded: missing insulin/vitamin D data. | 6026 | 2875 (46%) | ____ | HOMA-IR/22.5 | ≥2.00 | 8 (low) |
| Castro (2023) | Brazil | Cross-sectional | Probabilistic | Employees 35-74 years Brazil. Excluded: diabetes, HTN, follow-up losses, CVD. | 4717 | 3160 (67%) | 48 | HOMA-IR/22.5 | >2.85 | 8 (low) |
| Saruarov (2023) | Kazakhstan | Cross-sectional | Non-probabilistic | University employees 27-69 years (2019-2020). Excluded: kidney disease, diabetes. | 427 | 302 (70.7%) | ____ | HOMA-IR/22.5 | ≥2.5 | 7 (low) |
| Li (2023) | United States | Cross-sectional | Probabilistic | NHANES >18 years (2009-2018). Excluded: <18 years, missing data. | 12388 | 6417 (51.8%) | 47.5 | HOMA-IR/22.5 | > 2.0 | 8 (low) |
| Guardado-Mendoza (2024) | Mexico | Cross-sectional | Non-probabilistic | Subjects 18-65 years without diabetes (Guanajuato). Excluded: medication affecting glucose, pregnancy. | 1470 | 35.20% | 41 | HOMA-IR (Not specified) | ≥2 | 7 (low) |
| Vera-Ponce (2024) | Peru | Cross-sectional | Probabilistic | Adults >30 years without mental illness/pregnancy with complete variables. | 976 | 516 (52.87%) | ____ | HOMA-IR/22.5 | ≥ 2.8 | 8 (low) |
| Lee (2023) | South Korea | Cohort | Probabilistic | Adults 40-69 years. Excluded: missing HOMA-IR/CVD/mortality data. | 3597 | 1833 (51%) | 52.2 | HOMA-IR (Not specified) | ≥ 2.5 | 8 (low) |
| Song (2024) | United States | Cross-sectional | Probabilistic | NHANES 1999-2020. Excluded: <20 years, pregnancy, missing data. | 21,304 | 51.30% | 47.05 | HOMA-IR/22.5 | > 3.94 | 8 (low) |
| Wu (2024) | United States | Cross-sectional | Probabilistic | Non-diabetics ≥20 years. Excluded: pregnancy, non-morning sessions, without fasting, incomplete data. | 1,687 | 908 (52%) | 46.3 | HOMA-IR/22.5 | ≥2.6 | 8 (low) |

Supplementary Material 3. Forest plot of individual studies showing insulin resistance prevalence
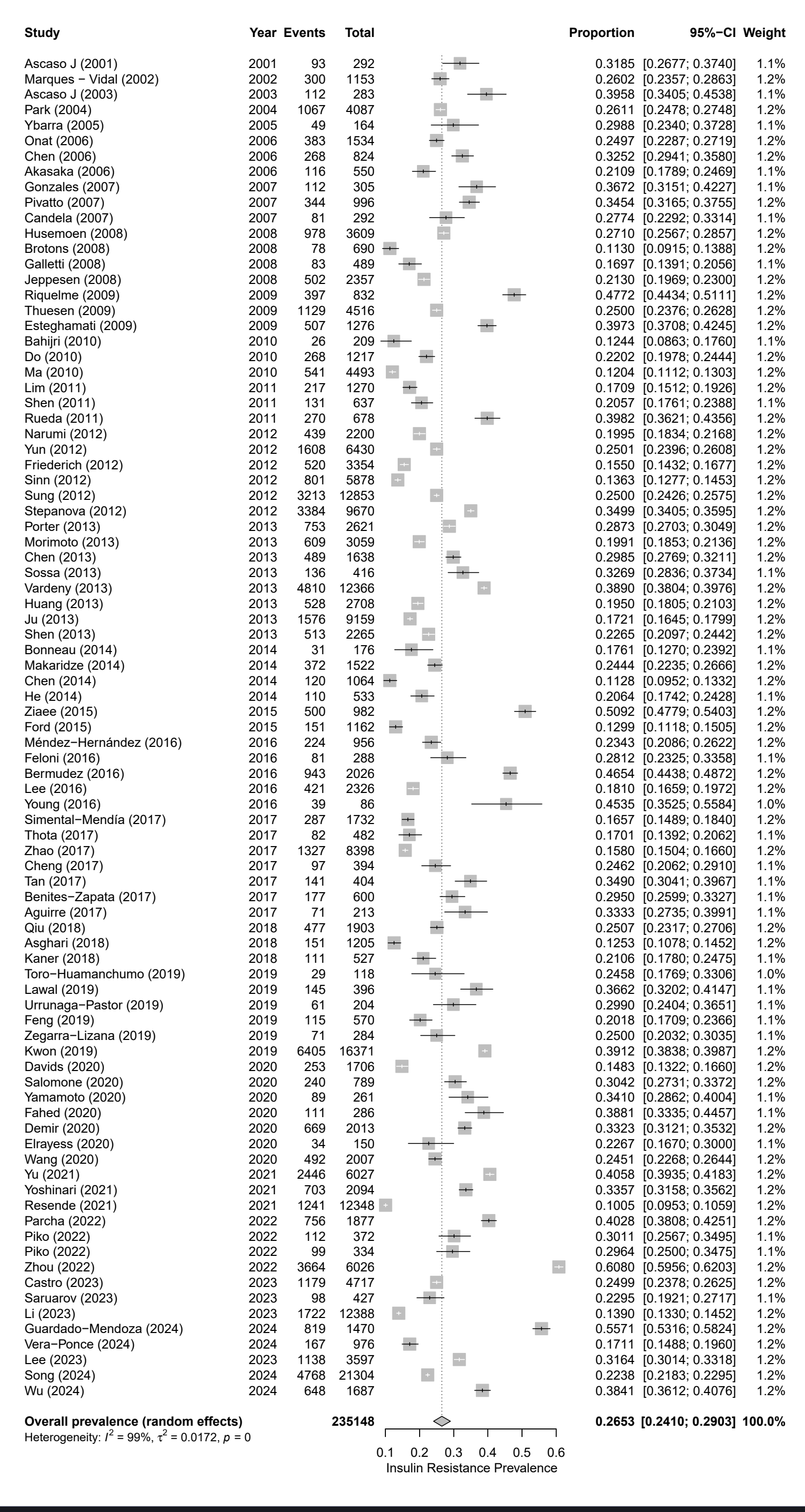


Supplementary Material 4. Funnel plot for assessment of publication bias.


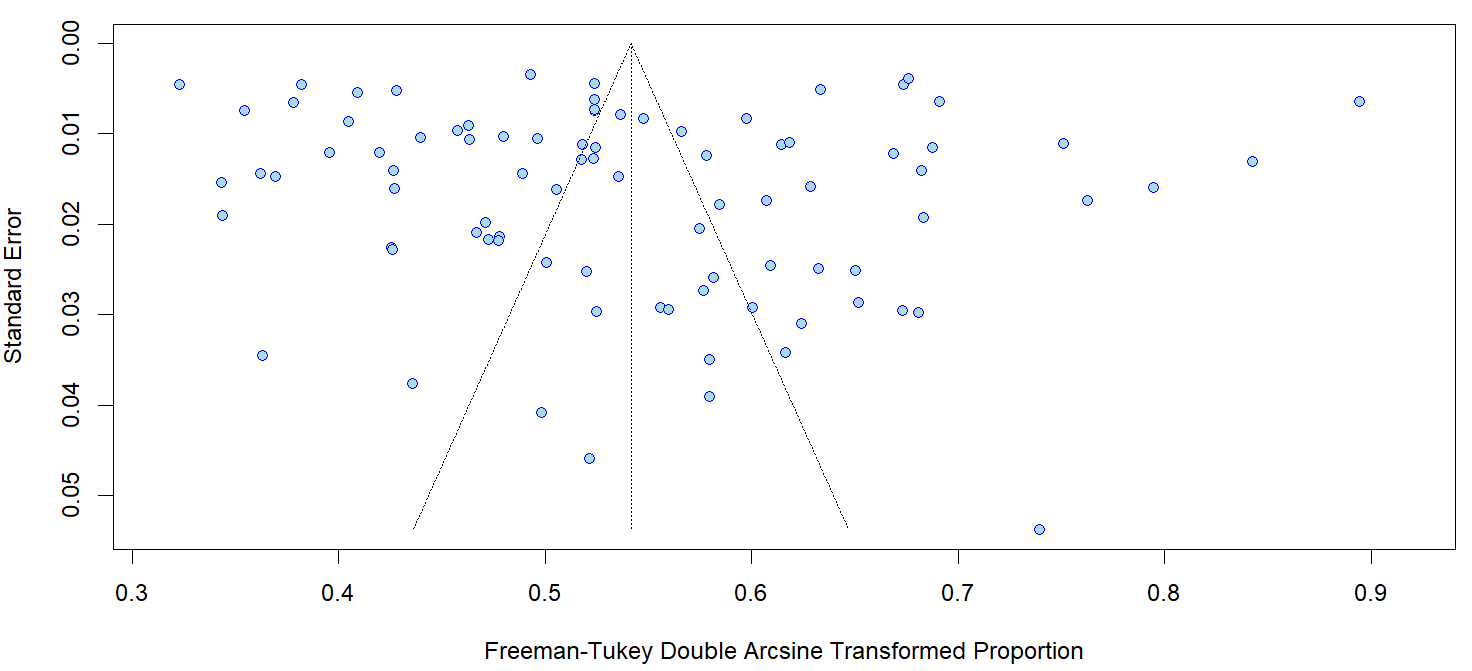

Supplement: Supplementary file 1 [file DataSheet1.docx]
